# Aerobic exercise improves VCI through circRIMS2/miR-186/BDNF-mediated neuronal apoptosis

**DOI:** 10.1186/s10020-020-00258-z

**Published:** 2021-01-07

**Authors:** Yali Niu, Chunxiao Wan, Jing Zhang, Shu Zhang, Zilong Zhao, Leshan Zhu, Xiaodong Wang, Xiaodong Ren, Jing Wang, Ping Lei

**Affiliations:** 1 Rehabilitation, The General Hospital, Tianjin Medical University, Tianjin, 300052 China; 2 Medical Imaging Department, The General Hospital, Tianjin Medical University, Tianjin, 300052 China; 3 Institute of Neurology, The General Hospital, Tianjin Medical University, Tianjin, 300052 China; 4 Neurosurgery, The General Hospital, Tianjin Medical University, Tianjin, 300052 China; 5 Department of Geriatrics, Tianjin Medical University General Hospital, Tianjin Geriatrics Institute, 154 Anshan Rd., Tianjin, 300052 People’s Republic of China

**Keywords:** circRIMS2, miR-186, BDNF, VCI, Aerobic exercise, Apoptosis

## Abstract

**Background:**

Vascular cognitive impairment (VCI) is a common cognitive disorder caused by cerebrovascular disease, ranging from mild cognitive impairment to dementia. Studies have shown that aerobic exercise might alleviate the pathological development of VCI, and our previous study observed that aerobic exercise could alleviate VCI through NF-κB/miR-503/BDNF pathway. However, there are few studies on the mechanism. Therefore, it is of great significance to fill the gaps in the mechanism for the early diagnosis of VCI and the clinical prevention and treatment of vascular dementia.

**Methods:**

CircRNA microarray analysis and quantitative real-time PCR were used to detect the expression of circRNA regulating synaptic be exocytosis 2 (RIMS2) (circRIMS2). Cell apoptosis was determined by TdT-mediated dUTP nick-end labeling (TUNEL) assay. The dual-luciferase reporter assay was performed to verify the interaction between circRIMS2 and miR-186, as well as miR-186 and BDNF. RNA pull-down assay detected the binding between circRIMS2 and miR-186. A VCI mouse model was established by repeated ligation of bilateral common carotid arteries (2VO). The lentiviral interfering vector was injected into the VCI mice through the lateral ventricle. The mice in the aerobic exercise group performed 30 min (12 m/min) running for 5 days a week. A Morris water maze test was performed after 4 weeks.

**Results:**

The expression of circRIMS2 and BDNF in the serum of VCI patients was significantly reduced, miR-186 expression was increased, and the expression of circRIMS2 was increased in the 2VO group of mice undergoing aerobic exercise. The expression levels of circRIMS2 and BDNF in the oxygen and glucose deprivation-treated (OGD-treated) cells were decreased, the miR-186 expression and cell apoptosis were increased, while the effect was weakened after transfection with the lentiviral vector pLO-ciR-RIMS2. CircRIMS2 could bind to miR-186, and after interference with circRIMS2 in HT22 cells, the expression of miR-186 was increased. Besides, miR-186 could bind to BDNF, and BDNF expression was decreased because of the overexpression of miR-186 in HT22 cells. The expression level of BDNF in the pLO-ciR-RIMS2 group was increased, and apoptosis was decreased, but the miR-186 mimic weakened the effect of pLO-ciR-RIMS2. Aerobic exercise could shorten the average time that mice reached the platform in the Morris water maze, increase the expression level of circRIMS2 and BDNF, reduce miR-186 expression, and inhibit neuronal apoptosis. However, the interference with circRIMS2 weakened this effect.

**Conclusion:**

The expression of circRIMS2 was down-regulated in VCI and aerobic exercise reduced neuronal apoptosis, and circRIMS2 improved VCI through the circRIMS2/miR-186/BDNF axis.

## Introduction

Vascular cognitive impairment (VCI) contains a series of cognitive impairments associated with cerebrovascular disease in a variety of ways and to varying degrees, ranging from mild non-dementia to vascular dementia (Flier et al. 2018). Until now, there is still a lack of effective methods of therapy. Research has shown that aerobic exercise could not only improve cardiovascular function but also improve the cognitive ability of patients with VCI (Liu-Ambrose et al. 2016; Ngandu et al. 2015). Recent studies have also suggested that aerobic exercise improves executive functions and neural efficiency in older patients with VCI (Hsu et al. 2018). Barha et al. found that S100B was involved in the effect of aerobic exercise on the alleviation of VCI (Barha et al. (2019)). Besides, our previous study found that aerobic exercise alleviated VCI via NF-κB/miR-503/BDNF axis (Niu et al. 2018). Our other study indicated that aerobic exercise alleviated VCI through the TUG1/BDNF axis (Wang et al. 2020). However, the mechanism by which aerobic exercise alleviates VCI remains rarely studied. So it is necessary to carry out more research on the mechanism of aerobic exercise to improve VCI.

Brain-derived neurotrophic factor (BDNF) is one of the key members of the neurotrophic factor family and is essential for the development of the nervous system (Chen and Chen 2017). BDNF can resist neuronal apoptosis induced by nerve injury. In the case of cerebral ischemia and hypoxia, BDNF can protect brain cells via inhibiting apoptosis (Chen et al. 2017; Xu et al. 2017). Our previous research has confirmed that aerobic exercise inhibited the apoptosis of hippocampal neurons through the NF-κB/miR-503/BDNF pathway, thereby alleviating the pathological development of VCI (Niu et al. 2018).

MicroRNAs affect our life activities such as cell growth, differentiation, and apoptosis. They also play an important role in the development of the nervous system (Kabekkodu et al. 2018). It is known that miR-186 is highly conserved in humans and other animals, and is closely correlative with neural function. In addition, it is reported that there was an intimate correlation between the expression of miR-186 and the pathological development of Alzheimer's disease (AD) (Satoh et al. 2015; Lau et al. 2013). However, the expression level and effect of miR-186 in VCI have not been reported. We have found that there are binding sites in the sequences of miR-186 and BDNF, suggesting that miR-186 might be involved in the pathology of VCI by regulating BDNF.

Circular RNA (circRNA) is a class of non-coding RNA molecules with a covalently formed ring structure and their role in the physiological and pathological processes of the nervous system has been reported in many studies (Chen and Yang 2015; Rossum et al. 2016; Floris et al. 2017). CircRNA regulating synaptic be exocytosis 2 (RIMS2) (circRIMS2) has been proved highly expressed in the human brain cortex and mouse brain tissue (Rybak-Wolf et al. 2015). Moreover, the bioinformatics software (circinteractome) predicted that there were 46 binding sites between miR-186 and circRIMS2, suggesting that there might be an interaction between miR-186 and circRIMS2. Therefore, we speculated that circRIMS2 might exert its neuroprotective effect by binding to miR-186 and inhibiting its expression.

Chronic exercise is known to play an important neuroprotective role in cerebral ischemia–reperfusion injury (IRI) (Dornbos and Ding 2012). The expression level of circRNA significantly changed in the brain tissues of oxygen and glucose deprivation (OGD) induced IRI, indicating that circRNA expression was closely related to the pathological development of brain IRI (Lin et al. 2016). It was speculated that aerobic exercise might increase circRIMS2 expression levels in VCI patients.

Therefore, we speculated that circRIMS2 was down-regulated in VCI, while aerobic exercise increased circRIMS2 expression level, and decreased the expression of miR-186, thus upregulated the expression level of BDNF protein, which affected the apoptosis of hippocampus neurons, and inhibited the pathological development of VCI and ultimately improved cognitive function.

## Materials and methods

### Human samples

Fasting venous blood samples were obtained from 20 patients with ACI and 20 healthy individuals from 9 to 11 a.m. Protocols used in all experiments were approved by the Tianjin Medical University General Hospital. We obtained informed consent from all donors before sample collection.

### Animals

All animal procedures used in this study met the standard of the local Institutional Animal Care and Use Committee of the General Hospital, Tianjin Medical University. 6–7-week-old C57BL/6 male mice (weight 22–26 g) were purchased from Charles River (Beijing, China). All animals were kept in a constant 12 h /12 h cycle of light and darkness, free for food and water.

### Experimental design

To induce VCI in vivo, The mice were treated with bilateral common carotid artery (CCA) occlusion (cerebral hypoperfusion; 2-VO). The procedure was as follows: Mice were anesthetized with 10% chloral hydrate (400 mg/kg, intraperitoneal injection), and subsequently made a midline neck incision. The bilateral CCA was obtusely separated and clamped to cause cerebral ischemia for 10 min and reperfused for 20 min, repeatedly for 3 times. For the sham group, only CCA separation was performed, and the vessels were not clipped.

Lentiviral vector pLV-shRNA and pLV-sh-RIMS2 were designed, packaged, and purified by Geneseed Biotech (Guangzhou, China). Mice were injected with pLV-shRNA or pLV-sh-RIMS2 by bilateral ventricles. And mice with aerobic exercise received treadmill exercise for 30 min (12 m/min) every day, 5 days a week. The Morris water maze experiment was performed 4 weeks later.

### Cell culture

HT22 cells were purchased from ATCC (Manassas, VA, USA). Cells were transferred to a DMEM medium containing 10% fetal bovine serum and 1% penicillin/streptomycin at 37 °C with 5% CO_2_. Upon confluent of 70%-80%, the cells were synchronized with serum-free SFM medium for 24 h and then treated with or without oxygen and glucose deprivation (OGD) for 1 h.

### Cell transfection

As a pretreatment, HT22 cells were treated with pLV-shRNA, pLV-sh-RIMS2, miR-186 mimic or its negative control mimic NC for 48 h, pLO-ciR-RIMS2, pLO-ciR-Control, pLO-ciR-RIMS2 + mimic NC, pLO-ciR-RIMS2 + miR-186 mimic (Geneseed Biotech Co., Ltd.) for 72 h using Lipofectamine™ 3000 reagent (Life Technologies Corporation, Carlsbad, CA, USA) according to its instruction for use. Then cells were washed and treated with or without oxygen and glucose deprivation (OGD) for 1 h.

### CircRNA microarray analysis

RNA in serum was extracted by the Trizol reagent (Invitrogen). The acquired RNA samples were labeled and hybridized to a circRNA microarray chip (Arraystar Inc.) according to the manufacturer's protocols. The acquired slides were then washed, fixed, and scanned with an Agilent Scanner G2505C. The scanned image was imported into Agilent Feature Extraction software and the R software package was used for further data processing.

### Quantitative real-time PCR (qRT-PCR)

Total RNA from serum, cells, and hippocampus was extracted by the Trizol reagent (Invitrogen). Reverse Transcription Kit (Takara, Tokyo, Japan) was used for reversely transcribing RNA to cDNA. The acquired cDNA was then mixed with SYBR Green Master Mix (Thermo Fisher) and amplified on an ABI 7900-fast thermocycler (Applied Biosystems). Primers were designed and synthesized by Sangon Biotech (Shanghai, China). The relative expression levels of mRNA were calculated with the 2^−∆∆Ct^ method and normalized to β-actin.

### Western-blot analysis

Total proteins were extracted from serum, cells, and hippocampus using RIPA lysis buffer (Beyotime Biotechnology) with phenylmethanesulfonyl fluoride (PMSF, Beyotime Biotechnology). The acquired protein samples were then separated by SDS-PAGE and transferred into the polyvinylidene difluoride (PVDF) membrane (Millipore). After blocking with 5% skimmed milk, the membranes were incubated with the Anti-BDNF antibody (ab108319, 1:1000) and the Anti-β-actin antibody (ab8227) at 4 °C overnight. Then the membranes were incubated with a secondary antibody (1:1000, Cell Signaling Technology) for 2 h at room temperature and the proteins were visualized with ECL Plus Western Blotting Substrate (Thermo Fisher).

### ELISA

The blood was placed at room temperature for 1 h then the supernatant was collected by centrifugation at 3000 rpm for 10 min. BDNF levels in serum were quantified by Brain-Derived Neurotrophic Factor (BDNF) kit according to the manufacturer's instructions (CYT306, Sigma-Aldrich).

### TUNEL

Apoptosis of HT22 cells or mouse hippocampal tissue was detected with the TUNEL Apoptosis Assay Kit (Beyotime) according to the manufacturer’s instructions. After that, the cells and tissues were stained with DAPI (Beyotime) and placed for 3–5 min at room temperature. Then they were washed 3 times with PBS (Gibco). An inverted fluorescence microscope (Olympus Optical, Ltd., Tokyo, Japan) was used to observe the apoptosis.

### Dual-luciferase reporter assay

The interaction between miR-186 and circRIMS2 was examined with a dual-luciferase reporter assay. The entire circRIMS2 sequence was cloned into the downstream region of the luciferase gene, denoted LUC-cRIMS2, while the mutant sequence was denoted with LUC-cRIMS2-mutant. Co-transfection of miR-186 mimic or mimic NC and LUC-cRIMS2 or LUC-cRIMS2-mutant into HEK 293T cells were performed. After 48 h, the luciferase activity was examined by the Dual-Luciferase Reporter Assay System (Promega, Madison, WI) according to the manufacturer's instructions.

The interaction between miR-186 and BDNF was examined with a dual-luciferase reporter assay. The WT-BDNF 3′-UTR or mutant-BDNF 3′-UTR was inserted into the pmirGLO vector and they were co-transfected into HEK-293 cells with miR-186 mimic or mimic NC. The luciferase activity was examined by the Dual-Luciferase Reporter Assay System (Promega, Madison, WI) according to the manufacturer's instructions.

### RNA pull-down assay

The biotinylated DNA probe complementary of miR-186 mimic and mimic NC were synthesized (Genepharma) and transfected into HEK-293 T cells. The cells were collected after 48 h. RNA pull-down assays were performed using the Pierce Magnetic RNA–Protein Pull-Down Kit (Thermo Fisher Scientific) according to the manufacturer's protocols. The expression level of circRIMS2 in the complex was detected by qRT–PCR analysis.

### Morris water maze

The Morris water maze was used to assess cognitive ability (Snow et al. 2015). The maze consisted of a 100 cm-diameter circular pool filled with water of 25 °C. There was an escape platform with a diameter of 7 cm in the center of the designated target quadrant, about 5 mm below the water level. Then, the mice were trained and given 60 s to find the platform 4 times a day for 6 days. After that, skimmed milk powder was added to make the water opaque. The time that mice found the underwater platform was recorded. After the Morris water maze was completed, the mice were sacrificed and hippocampal tissues were collected.

### Statistical analysis

All data were presented as mean ± standard deviation using SPSS 22.0 (Chicago, IL, USA). Student’s t-test or one-way ANOVA was used to evaluate the statistical significance between different groups. A value of *P* < 0.05 was considered statistically significant. The sample size was calculated under the condition (α = 0.05, two-tailed and a power of 80%).

## Results

### The down-regulation of circRIMS2 and BDNF in the serum of VCI patients

**Fig. 1:**
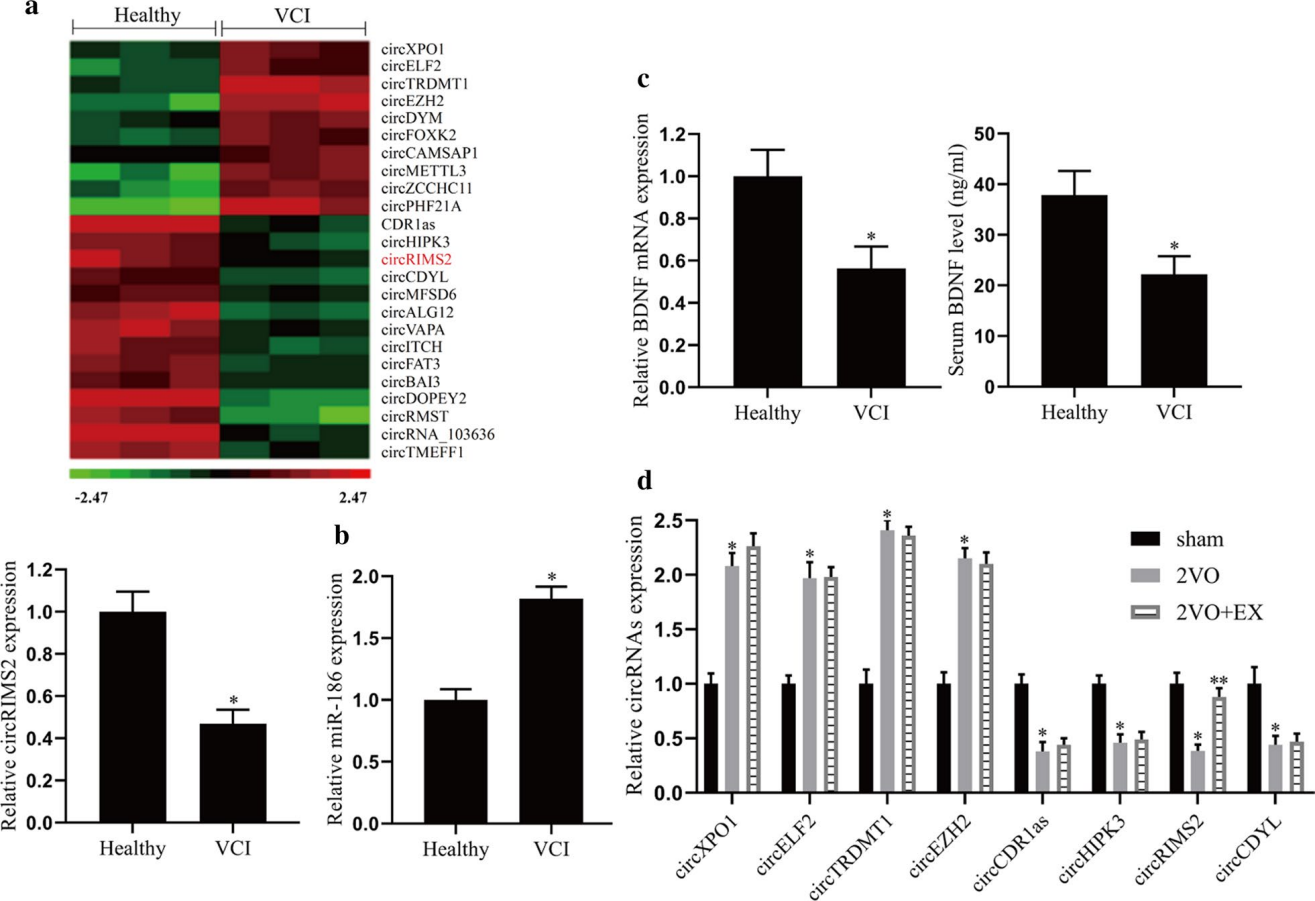
Expression of circRIMS2 and BDNF protein in the serum of VCI patients. Fasting venous blood from healthy individuals (n = 20) and VCI patients (n = 20) were collected and centrifuged to obtain serum. **a** CircRNA microarray analysis and qRT-PCR were used to detect the expression level of circRIMS2 in serum. **b** qRT-PCR detected the expression level of miR-186. **c** qRT-PCR detected the mRNA expression level of BDNF, and ELISA detected the BDNF protein content in serum. **d** Repeated ligation of bilateral common carotid arteries (CCA) caused ischemia–reperfusion injury (2VO) to establish a VCI mouse model and the mice were divided into three groups (n = 6 per group): sham, 2VO, 2VO + aerobic exercise (EX), and qRT-PCR detected the expression of circ-RNAs. *P < 0.05 versus the Healthy group or sham group; **P < 0.01 versus the 2VO group

Fasting venous blood from healthy people (n = 20) and VCI patients (n = 20) were collected, and centrifuged to separate the serum. CircRNA microarray analysis and qRT-PCR detected the expression of circRIMS2 in serum and found that compared with healthy individuals, the expression level of RIMS2 in the serum of VCI patients was significantly reduced (Fig. 1a). The result of qRT-PCR showed that miR-186 expression in the serum of VCI patients was significantly increased (Fig. 1b). Meanwhile, qRT-PCR and ELISA detected the mRNA levels and protein content of BDNF respectively, the results showed that compared with healthy people, the expression of BDNF was decreased in VCI patients (Fig. 1c). According to the results of RNA chip detection, we screened eight circRNAs that had significant changes in the serum of VCI patients. Then the expression levels of these circRNAs in the hippocampus of the mice were detected by qRT-PCR. The result showed that the expression of circRIMS2 in the 2VO group was decreased, while aerobic exercise increased the expression of circRIMS2 in 2VO mice, and aerobic exercise had no significant effect on the expression of the other circRNAs (Fig. 1d).

### Overexpression of circRIMS2 inhibited the apoptosis of hippocampal neurons induced by OGD treatment

**Fig. 2:**
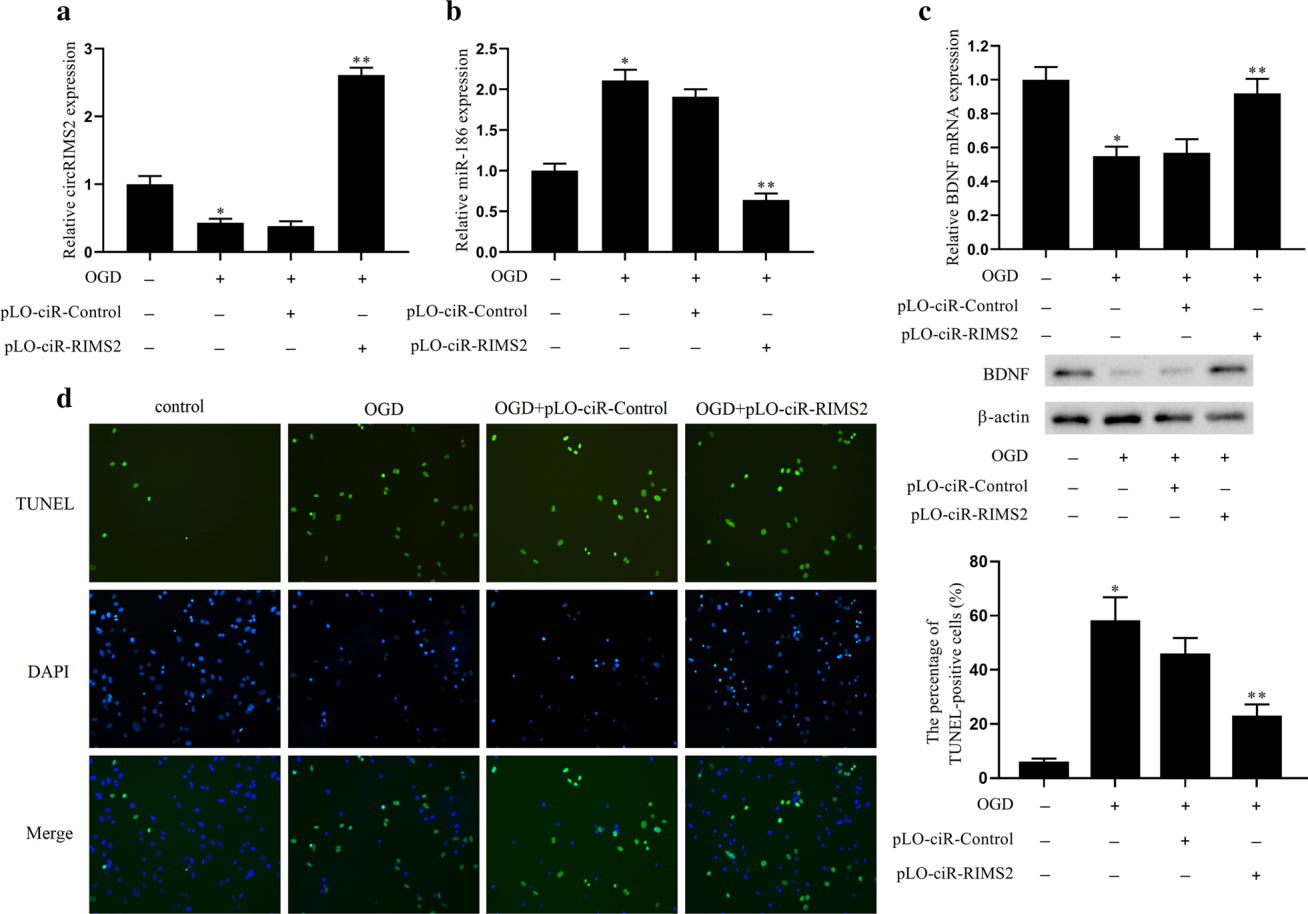
Overexpression of circRIMS2 affects the apoptosis of hippocampal neurons. The lentiviral vector pLO-ciR-Control or pLO-ciR-RIMS2 was transfected into HT22 cells for 72 h, then treated with oxygen and glucose deprivation (OGD) for 1 h and grouped: control, OGD, OGD + pLO-ciR-Control, OGD + pLO-ciR-RIMS2. **a** qRT-PCR detected the expression of circRIMS2 in cells. **b** qRT-PCR detected the expression of miR-186. **c** BDNF mRNA level was detected by qRT-PCR, and the BDNF protein level was determined by western blot. **d** TUNEL detected cell apoptosis. *P < 0.05 versus the control group; **P < 0.01 versus the OGD + pLO-ciR-Control group

Next, explore the effect of circRIMS2 on the apoptosis of hippocampal neurons. The lentiviral vector pLO-ciR-Control or pLO-ciR-RIMS2 was transfected into mouse hippocampal neuron cells HT22 for 72 h, then treated with oxygen and glucose deprivation (OGD) for 1 h and divided into four groups: control, OGD, OGD + pLO-ciR-Control, OGD + pLO-ciR-RIMS2. The result showed that the expression of circRIMS2 and BDNF was decreased in OGD, while the effect was reversed after transfection with the lentiviral vector pLO-ciR-RIMS2 (Fig. 2a, c). The expression of miR-186 was increased, and cell apoptosis was increased in OGD, while the effect was reversed after transfection with the lentiviral vector pLO-ciR-RIMS2 (Fig. 2b, d). These results suggested overexpression of circRIMS2 could improve the BDNF level, decrease the expression of miR-186, and inhibited the apoptosis of hippocampal neurons induced by OGD treatment.

### The interaction between circRIMS2 and miR-186 and the regulatory effect of miR-186 on BDNF expression

**Fig. 3:**
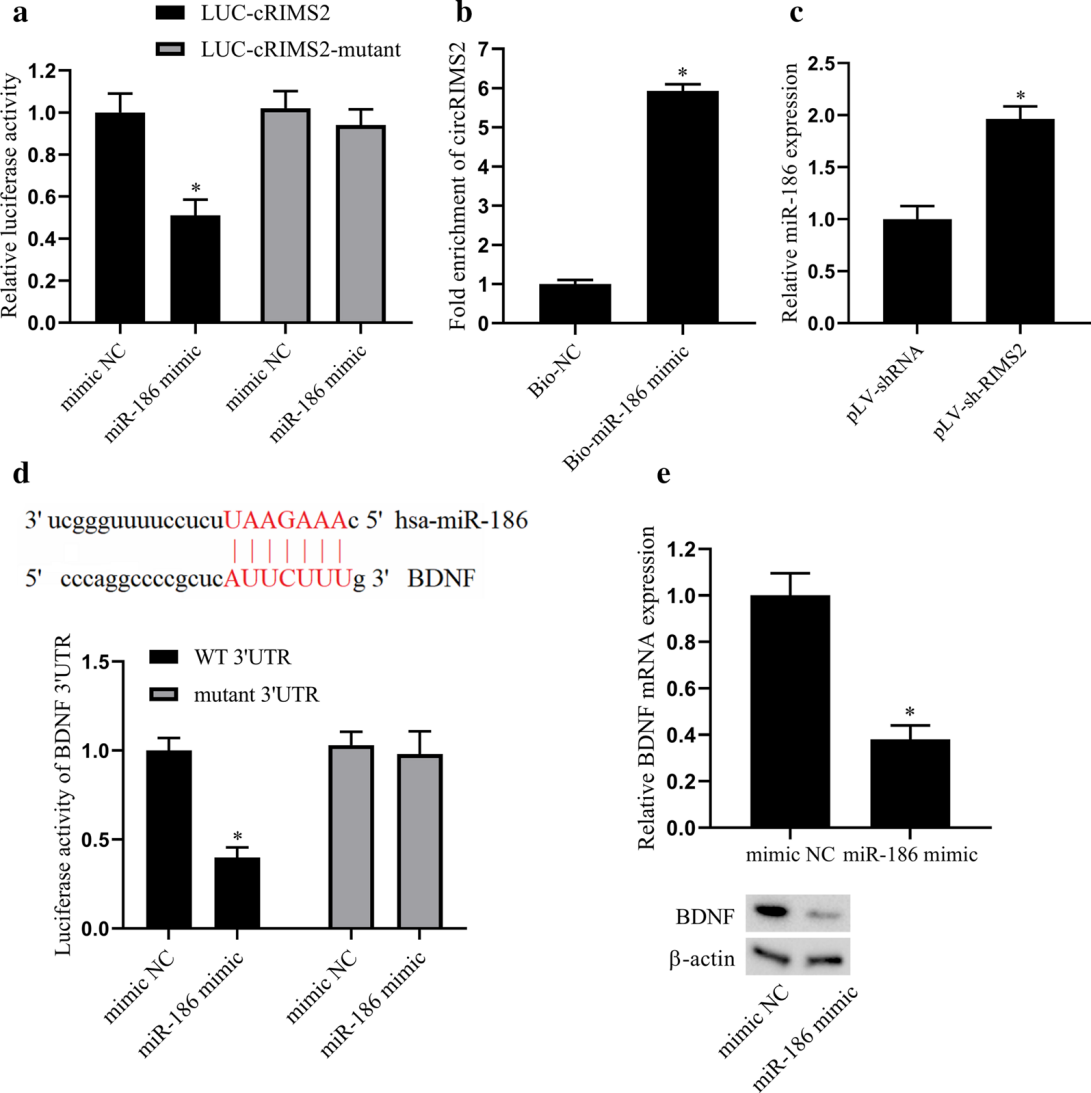
The interaction between circRIMS2 and miR-186 and the regulatory effect of miR-186 on BDNF expression. **a** The luciferase reporter gene test was performed in HEK-293T cells to detect the binding and interaction between circRIMS2 and miR-186. **b** RNA pull-down detected the binding of circRIMS2 and miR-186. **c** HT22 cells were transfected with the lentiviral vector pLV-shRNA or pLV-sh-RIMS2 for 48 h, and the expression level of miR-186 was detected by qRT-PCR. **d** Bioinformatics predicted the binding sites of miR-186 and BDNF, and the luciferase reporter gene test in HEK-293T cells detected the regulatory effect of miR-186 on BDNF. **e** HT22 cells were transfected with miR-186 mimic or its negative control mimic NC for 48 h, the BDNF mRNA level was detected by qRT-PCR, and the BDNF protein level was determined by western blot. *P < 0.05 versus mimic NC or Bio-NC or pLV-shRNA

Bioinformatics analysis of the binding sites of circRIMS2 and miR-186 (Table 1). In HEK-293T cells, the luciferase test detected the interaction between circRIMS2 and miR-186. The result showed that after miR-186 overexpressed, the luciferase activity in LUC-cRIMS2 transfected cells was inhibited, but there was no change in the LUC-cRIMS2-mutant group (Fig. 3a). The result of RNA pull-down indicated the binding of circRIMS2 to miR-186 (Fig. 3b). HT22 cells were then transfected with the lentiviral vector pLV-shRNA or pLV-sh-RIMS2 for 48 h, the expression level of miR-186 was detected by qRT-PCR. The result indicated that the expression of miR-186 increased after interference with the circRIMS2 in HT22 cells (Fig. 3c). Bioinformatics predicted the binding sites of miR-186 and BDNF, and luciferase test was performed to detect the regulatory effect of miR-186 on BDNF in HEK-293T cells. The result indicated that the luciferase activity in BDNF WT 3′UTR group was inhibited after transfected with miR-186 mimic, but there was no significant change in the mutant 3′ UTR group (Fig. 3d). HT22 cells were transfected with miR-186 mimic or mimic NC for 48 h, the mRNA expression level of BDNF was detected by qRT-PCR, and the expression of BDNF protein level was determined by western blot. The results showed that the expression of BDNF was decreased after transfected with miR-186 mimic (Fig. 3e). Taken together, our results indicated that circRIMS2 had a negative regulatory effect on miR-186 and miR-186 negatively regulated BDNF as well.

**Table 1:** The predicted binding sites of circRIMS2 (HSA_circ_0005114) and miR-186

CircRNA Mirbase ID	CircRNA(top)-miRNA (Bottom) pairing	CircRNA(top)-miRNA (Bottom) pairing	CircRNA(top)-miRNA (Bottom) pairing
hsa circ 0005114 (5′…3′) hsa-miR-186(3′…5′)	AAAUAGAACAGAUACAUUCUUUC ||||||| UCGGGUUUUCCUCUUAAGAAAC	UUUGUUGUUGUUUUCUUCUUUAU |||||| UCGGGUUUUCCUCUUAAGAAAC	UAUUCAAUAAAUUCU–UUCUUUAG ||| |||||| UCGGGUUUUCCUCUUAAGAAAC
hsa circ 0005114 (5′…3′) hsa-miR-186(3′…5′)	AUUCAGUUGUAAUUUUUCUUUAG |||||| UCGGGUUUUCCUCUUAAGAAAC	UGUUCCAUUUAUUUUUUCUUUAU |||||| UCGGGUUUUCCUCUUAAGAAAC	GAAAUUGAGUUGGAUUUCUUUAC |||||| UCGGGUUUUCCUCUUAAGAAAC
hsa circ 0005114 (5′…3′) hsa-miR-186(3′…5′)	AGAAACAGCUAAUAGUUCUUUAG |||||| UCGGGUUUUCCUCUUAAGAAAC	GAAAACAUUUAUUUUUUCUUUAC |||||| UCGGGUUUUCCUCUUAAGAAAC	GAUGGAAUAUUCAUGUUCUUUAU |||||| UCGGGUUUUCCUCUUAAGAAAC
hsa circ 0005114 (5′…3′) hsa-miR-186(3′…5′)	AGAAACAGCUAAUAGUUCUUUAG |||||| UCGGGUUUUCCUCUUAAGAAAC	GCCACCAUACCCAGCUUCUUUAU |||||| UCGGGUUUUCCUCUUAAGAAAC	AAAUGAUAGGAUUUCAUUCUUUU |||| |||||| UCGGGUUUUCCUCU-UAAGAAAC
hsa circ 0005114 (5′…3′) hsa-miR-186(3′…5′)	CAUCUAUAUAUCUAUAUUCUUUU ||||||| UCGGGUUUUCCUCUUAAGAAAC	UACUGUCCAAAAUUU-AUUCUUUG |||||| ||||||| UCGGGUUUUCCUCUUAAGAAAC	UGUAUGUACCACAUUUUCUUUAU |||||| UCGGGUUUUCCUCUUAAGAAAC
hsa circ 0005114 (5′…3′) hsa-miR-186(3′…5′)	AUCAAUUAGAUAAAUAUUCUUUA ||||||| UCGGGUUUUCCUCUUAAGAAAC	UUAGCUUAUAUUUACAUUCUUUA ||||||| UCGGGUUUUCCUCUUAAGAAAC	AUUAGAGAAAAUUCC-UUCUUUAU |||| |||||| UCGGGUUUUCCUCUUAAGAAAC
hsa circ 0005114 (5′…3′) hsa-miR-186(3′…5′)	AUCAAUUAGAUAAAUAUUCUUUA ||||||| UCGGGUUUUCCUCUUAAGAAAC	CAAACUUUGGCCUCUAUUCUUUC ||||||| UCGGGUUUUCCUCUUAAGAAAC	AUACGUUUAAAAUUU-UUCUUUAA ||| |||||| UCGGGUUUUCCUCUUAAGAAAC
hsa circ 0005114 (5′…3′) hsa-miR-186(3′…5′)	AUUCUUAGUGACGGUUUCUUUAU |||||| UCGGGUUUUCCUCUUAAGAAAC	CUACCUGCCUAUAACUUCUUUAA |||||| UCGGGUUUUCCUCUUAAGAAAC	UUUCUCAGUUUUCUCUUCUUUAA |||||| UCGGGUUUUCCUCUUAAGAAAC
hsa circ 0005114 (5′…3′) hsa-miR-186(3′…5′)	AUAUGAAACAGUUAGAUUCUUUU ||| ||||||| UCGGGUUUUCCUCU–UAAGAAAC	UUUAAAAAAACAUAUUUCUUUAU |||| |||||| UCGGGUUUUCCUCUUAAGAAAC	UAGUACUUAUAUCAGAUUCUUUA ||||||| UCGGGUUUUCCUCUUAAGAAAC
hsa circ 0005114 (5′…3′) hsa-miR-186(3′…5′)	UUAAAAUCAGUGUGGAUUCUUUU ||||||| UCGGGUUUUCCUCUUAAGAAAC	GUUGACAGCUUUGUCAUUCUUUU ||||||| UCGGGUUUUCCUCUUAAGAAAC	CAUUUCUUACUGGAAAUUCUUUA ||||||| UCGGGUUUUCCUCUUAAGAAAC
hsa circ 0005114 (5′…3′) hsa-miR-186(3′…5′)	GUUGUCCGGAAAAACUUCUUUAC |||||| UCGGGUUUUCCUCUUAAGAAAC	UCCCAGGUUCAAGCA-AUUCUUUU ||| ||||||| UCGGGUUUUCCUCUUAAGAAAC	UAAUUUAUUCAAUAAAUUCUUUC |||||| UCGGGUUUUCCUCUUAAGAAAC
hsa circ 0005114 (5′…3′) hsa-miR-186(3′…5′)	AAAAAUAUUUUUCGUAUUCUUUC ||||||| UCGGGUUUUCCUCUUAAGAAAC	CAAAUAAAGCCUGUCUUCUUUAG |||| |||||| UCGGGUUUUCCUCUU-AAGAAAC	ACUAGAUUUGCCUUUUUCUUUAA |||||| UCGGGUUUUCCUCUUAAGAAAC
hsa circ 0005114 (5′…3′) hsa-miR-186(3′…5′)	GCUAAUUUCUCUUCUUUCUUUAU |||||| UCGGGUUUUCCUCUUAAGAAAC	GUUUAUUAAGUAAUUAUUCUUUA ||| ||||||| UCGGGUUUUCCUCU-UAAGAAAC	UGCCCAUAAUUACAAAUUCUUUA ||||||| UCGGGUUUUCCUCUUAAGAAAC
hsa circ 0005114 (5′…3′) hsa-miR-186(3′…5′)	AGCCUUAGAAAUAAU-UUCUUUAC ||| |||||| UCGGGUUUUCCUCUUAAGAAAC	GAAUUACUGUAUAUCUUCUUUAU |||||| UCGGGUUUUCCUCUUAAGAAAC	UUGAUUUUGGUCAAGUUCUUUAU |||||| UCGGGUUUUCCUCUUAAGAAAC

Displayed 42 binding sites among the total 46 sites

### CircRIMS2 inhibits neuronal apoptosis through the miR-186/BDNF pathway

**Fig. 4:**
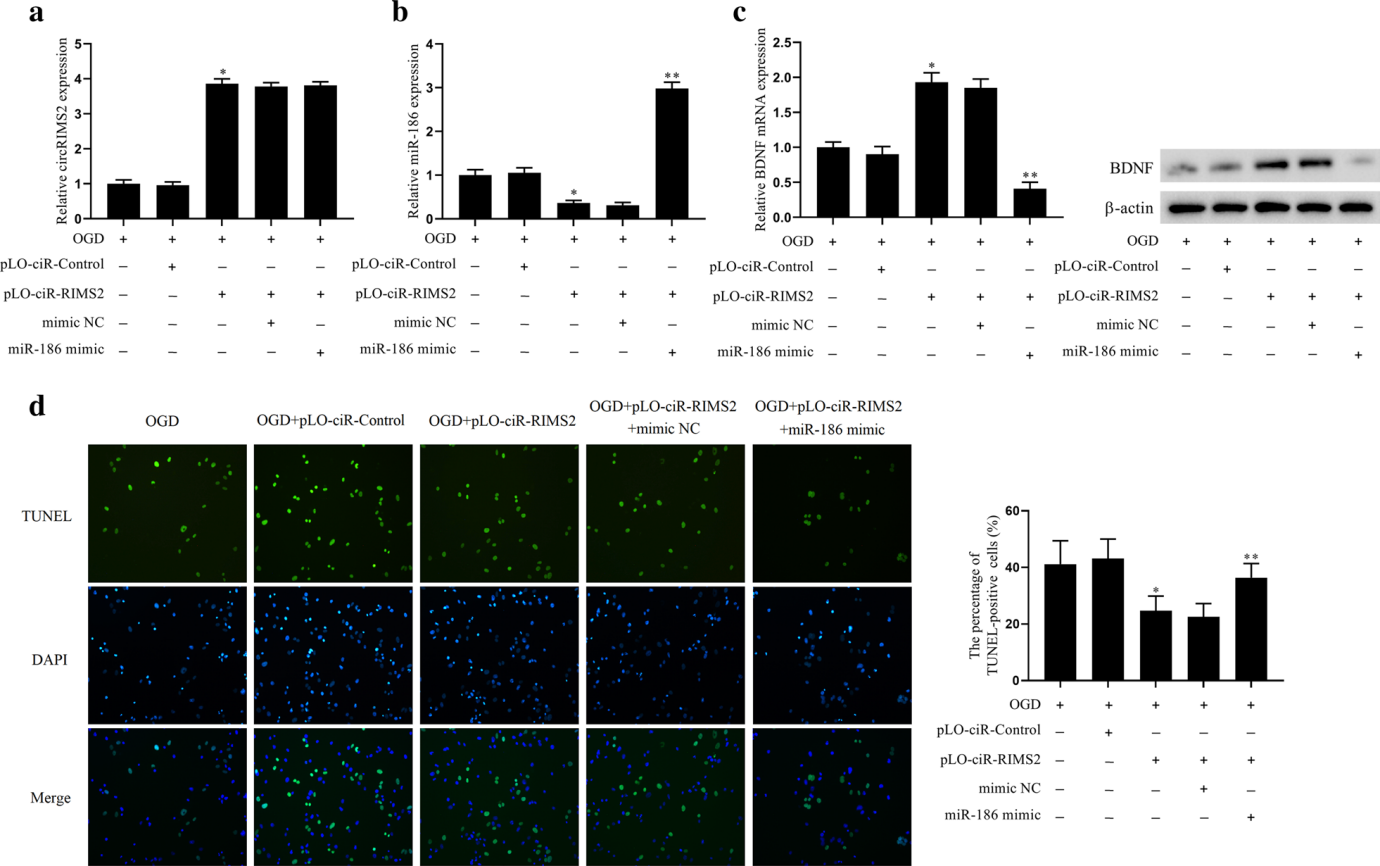
CircRIMS2 inhibits neuronal apoptosis through the miR-186/BDNF pathway. The lentiviral vector pLO-ciR-Control, pLO-ciR-RIMS2, pLO-ciR-RIMS2 + mimic NC, or pLO-ciR-RIMS2 + miR-186 mimic was transfected into HT22 cells for 72 h, then treated with oxygen and glucose deprivation (OGD) for 1 h and grouped: OGD, OGD + pLO-ciR-Control, OGD + pLO-ciR-RIMS2, OGD + pLO-ciR-RIMS2 + mimic NC, OGD + pLO-ciR-RIMS2 + miR-186 mimic. **a** qRT-PCR detected the expression of circRIMS2 in cells. **b** qRT-PCR detected the expression of miR-186. **c** BDNF mRNA level was detected by qRT-PCR, and the BDNF protein level was determined by western blot. **d** TUNEL detected cell apoptosis. *P < 0.05 versus OGD + pLO-ciR-Control; **P < 0.01 versus OGD + pLO-ciR-RIMS2 + mimic NC

Since we had found overexpression of circRIMS2 could inhibit the apoptosis and proved the relationship between circRIMS2 and miR-186, as well as the regulatory effect of miR-186 on BDNF expression, we next investigated whether circRIMS2 inhibited neuronal apoptosis through the miR-186/BDNF pathway. The lentiviral vector pLO-ciR-Control, pLO-ciR-RIMS2, pLO-ciR-RIMS2 + mimic NC, or pLO-ciR-RIMS2 + miR-186 mimic was transfected into HT22 cells for 72 h, then treated with oxygen and glucose deprivation (OGD) for 1 h and grouped: OGD, OGD + pLO-ciR-Control, OGD + pLO-ciR-RIMS2, OGD + pLO-ciR-RIMS2 + mimic NC, OGD + pLO-ciR-RIMS2 + miR-186 mimic. The result of qRT-PCR showed that the expression of circRIMS2 and BDNF was decreased in OGD, while the effect was reversed after transfected with the lentiviral vector pLO-ciR-RIMS2. However, compared with OGD + pLO-ciR-RIMS2, the expression was decreased after transfected with the lentiviral vector pLO-ciR-RIMS2 + miR-186 mimic (Fig. 4a, c). The expression of miR-186 and cell apoptosis were increased in OGD, while this effect was reversed after transfected with the lentiviral vector pLO-ciR-RIMS2. However, compared with OGD + pLO-ciR-RIMS2, the expression was increased after transfected with the lentiviral vector pLO-ciR-RIMS2 + miR-186 mimic (Fig. 4b, d). These results confirmed our previous hypothesis that CircRIMS2 inhibited neuronal apoptosis through the miR-186/BDNF pathway.

### Aerobic exercise affected the cognitive function of VCI model mice through the circRIMS2/BDNF pathway in vivo

**Fig. 5:**
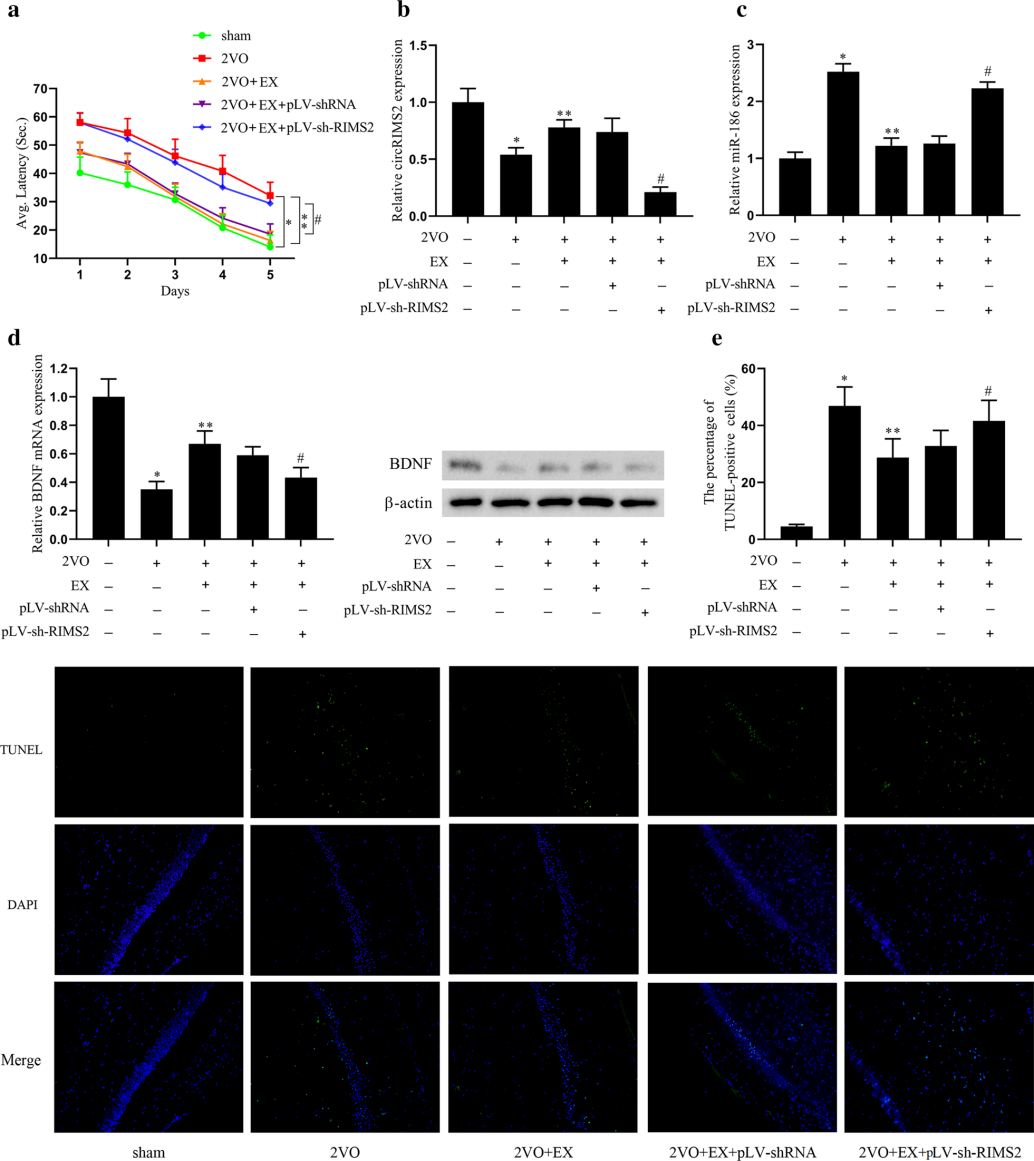
Aerobic exercise affects the cognitive function of the VCI model through the circRIMS2/BDNF pathway in vivo. The lentiviral vector pLV-shRNA or pLV-sh-RIMS2 was injected into VCI model mice through lateral ventricle. Repeated ligation of bilateral common carotid arteries (CCA) caused ischemia–reperfusion injury (2VO) to establish a VCI model. The mice were grouped (n = 6 per group): sham, 2VO,2VO + EX,2VO + EX + pLV-shRNA, and 2VO + EX + pLV-sh-RIMS2. **a** The water maze tested the time that mice found the underwater platform and calculate the average latency. **b** qRT-PCR was used to detect the expression of circRIMS2 in hippocampus. **c** qRT-PCR detected the expression of miR-186. **d** BDNF mRNA level was detected by qRT-PCR, and the BDNF protein level was determined by western blot. **e** TUNEL detected apoptosis of hippocampal cells. *P < 0.05 versus the sham group; **P < 0.01 versus the 2VO group; ^#^*P* < 0.05 versus the 2VO + EX + pLV-shRNA group

In the following work, we tested whether aerobic exercise affected the cognitive function of VCI model mice through the circRIMS2/BDNF pathway in vivo. The lentiviral vector pLV-shRNA or pLV-sh-RIMS2 was injected into VCI model mice through lateral ventricle. Repeated ligation of bilateral common carotid arteries (CCA) caused ischemia–reperfusion injury to establish a VCI mouse model. The mice were grouped (n = 6 per group): sham, 2VO, 2VO + EX, 2VO + EX + pLV-shRNA, and 2VO + EX + pLV-sh-RIMS2. The Morris water maze test indicated that the time to find the underwater platform in 2VO was significantly increased compared with the sham group while aerobic exercise decreased the time. However, the platform latency was increased after transfected with 2VO + EX + pLV-sh-RIMS2 (Fig. 5a). Then we determined the expression of circRIMS2 and BDNF, found that they were decreased in the 2VO group while aerobic exercise could improve the expression but interference of RIMS2 could reduce this improvement (Fig. 5b, d). We also detected miR-186 expression and apoptosis in the hippocampus, which proved that aerobic exercise could decrease the expression of miR-186 and inhibited the apoptosis of hippocampal neurons induced by VCI, and interference of RIMS2 could reverse the weakening of miR-186 expression and apoptosis (Fig. 5c, e). These results demonstrated that aerobic exercise upregulated the expression of circRIMS2, resulting in the down-regulation of miR-186, which upregulated the expression of BDNF, reduced the apoptosis of hippocampal neurons, inhibited the pathological development of VCI, and finally improved the cognitive function.

## Discussion

Vascular cognitive impairment (VCI) is a form of a syndrome caused by cerebrovascular diseases ranging from mild cognitive impairment to dementia, and vascular dementia is the most serious form of VCI (Flier et al. 2018; Iadecola et al. 2019). Up to now, there is still a lack of unified diagnostic criteria and treatment. Studies have shown that sustained aerobic exercise could not only improve cardiovascular function but also improve the cognitive ability of elderly patients with VCI (Liu-Ambrose et al. 2016; Ahlskog et al. 2011). In this study, we found that circRIMS2 was decreased in VCI models, and aerobic exercise could increase the expression level of circRIMS2. After aerobic exercise, miR-186 was down-regulated, resulting in the increase of BDNF, and further reducing the apoptosis of hippocampal neurons, thereby inhibiting the pathological development of VCI and improving cognitive functions.

Studies indicated that aerobic exercise was positively associated with cognition and could improve age-related cognitive decline (Wang and Holsinger 2018; Brem and Sensi 2018). Brain-derived neurotrophic factor (BDNF) is a neurotrophic substance that promotes neuronal survival and synaptic integrity. BDNF is mainly expressed in brain regions such as the hippocampus and cortex and can promote memory storage and neuroprotection (Tapia-Arancibia et al. 2008; Zheng et al. 2012; Huang and Reichardt 2001). Evidence suggested that BDNF was a key mediator of aerobic exercise to improve cognitive function (Wang and Holsinger 2018). Here, we established a mouse VCI model by repeated ligation of bilateral common carotid arteries. Likewise, the BDNF expression level in serum of VCI patients and hippocampal tissues of mice was low, while it was increased in hippocampal tissues of mice after aerobic exercise. Then we used OGD to induce cerebral ischemia–reperfusion injury (IRI) in vitro and found that BDNF expression was decreased and apoptosis was increased after OGD treatment.

MicroRNAs are widely involved in the regulation of gene expression by binding to target gene mRNA, affecting important life activities such as cell growth, differentiation, apoptosis, and downstream signal transduction, and playing an important regulatory role in the occurrence and development of human diseases (Kabekkodu et al. 2018). For instance, MiR-26b has been reported to be up-regulated in Alzheimer's disease (AD) (Absalon et al. 2013). In addition, miR-186 is also closely related to neurological function. Studies have shown that chronic stress stimulation could lead to an increase of miR-186 in the hippocampus and prefrontal cortex of rats (Babenko et al. 2012). Satoh et al*.* have confirmed that the expression level of miR-186 in the blood of patients with AD was higher than that of normal people (Satoh et al. 2015), and the expression level of miR-186 was up-regulated in the prefrontal cortex of patients with AD (Lau et al. 2013), indicating that the expression level of miR-186 is closely related to the pathological development of AD. However, miR-186 has not been reported in VCI. In our study, the results showed that miR-186 was decreased after aerobic exercise compared with the VCI models. The bioinformatics analysis and the double-luciferase assay and plasmid transfection verified the interaction between miR-186 and BDNF, as well as the negative regulation of BDNF by miR-186.

CircRNA, an endogenous non-coding RNA, plays a role in the sponge effect of miRNA and inhibits its activity by binding to miRNA, thereby relieving the inhibition of miRNA on its target genes and leading to up-regulation of target gene expression (Hansen et al. 2013; Bu et al. 2019). CircRNA plays a crucial regulatory role in the occurrence and development of neurological diseases (Rossum et al. 2016; Floris et al. 2017). Studies have shown that circRNA Cdr1as could regulate miRNA levels in mammalian brains, while Cdr1as deletion could lead to abnormal neuronal activity and behavioral disorders in mice (Piwecka et al. 2017). CircRNA regulating synaptic be exocytosis 2 (RIMS2) (CircRIMS2) is a circRNA that is highly expressed in the neural tissue. Rybak-wolf et al*.* have demonstrated that it was highly expressed in the human cerebral cortex and mouse brain tissues, and has good conservatism in the process of neural differentiation (Rybak-Wolf et al. 2015). CircRNA microarray analysis and qRT-PCR showed that the expression of circRIMS2 was significantly lower than that of the control group in the serum of VCI patients, OGD-induced HT22 cell model, and in the hippocampus of mouse VCI model induced by repeated ligation of bilateral common carotid arteries. Besides, we proved the interaction between circRIMS2 and miR-186 and confirmed that circRIMS2 inhibited neuronal apoptosis through the miR-186 /BDNF pathway. Subsequently, we conducted in vivo experiments to verify that aerobic exercise affected the cognitive function of VCI model mice partly through the circRIMS2/BDNF axis.

## Conclusions

In conclusion, our study was the first to investigate the correlation between circRIMS2 expression level in serum and cognitive function of VCI patients and the effect of circRIMS2 on neuronal apoptosis and differentiation. Besides, our study combines the role of aerobic exercise and circRNAs in the pathological development of VCI for the first time, which not only further confirms the improvement effect of aerobic exercise on cognitive function, but also emphasizes the key role of circRNAs in it. This work is not only of great significance for the early diagnosis of VCI and the clinical prevention and treatment of vascular dementia but also will bring a breakthrough for the clinical diagnosis and treatment of AD.

However, our study is limited by the small sample size, as it is extremely difficult to recruit the subjects. In the following work, larger sample size will be taken to investigate.

## Data Availability

All data generated or analyzed during this study are included in this published article.
